# 21st century (clinical) decision support in nursing and allied healthcare. Developing a learning health system: a reasoned design of a theoretical framework

**DOI:** 10.1186/s12911-023-02372-4

**Published:** 2023-12-05

**Authors:** Mark van Velzen, Helen I. de Graaf-Waar, Tanja Ubert, Robert F. van der Willigen, Lotte Muilwijk, Maarten A. Schmitt, Mark C. Scheper, Nico L. U. van Meeteren

**Affiliations:** 1 Data Supported Healthcare: Data-Science unit, Research Center Innovations in care, Rotterdam University of Applied Sciences, Rotterdam, the Netherlands; 2 Department of Anesthesiology, Erasmus Medical Center, Rotterdam, the Netherlands; 3 Institute for Communication, media and information Technology, Rotterdam University of Applied Sciences, Rotterdam, the Netherlands; 4 Allied Health professions, faculty of medicine and science, Macquarrie University, Sydney, Australia; 5 Top Sector Life Sciences and Health (Health~Holland), The Hague, the Netherlands

**Keywords:** Learning health system, Clinical decision support system, Experience based evidence, Allied healthcare, Nursing, Functionomics, Personomics, Key enabling technologies, Key enabling methodologies

## Abstract

In this paper, we present a framework for developing a Learning Health System (LHS) to provide means to a computerized clinical decision support system for allied healthcare and/or nursing professionals. LHSs are well suited to transform healthcare systems in a mission-oriented approach, and is being adopted by an increasing number of countries. Our theoretical framework provides a blueprint for organizing such a transformation with help of evidence based state of the art methodologies and techniques to eventually optimize personalized health and healthcare. Learning via health information technologies using LHS enables users to learn both individually and collectively, and independent of their location. These developments demand healthcare innovations beyond a disease focused orientation since clinical decision making in allied healthcare and nursing is mainly based on aspects of individuals’ functioning, wellbeing and (dis)abilities. Developing LHSs depends heavily on intertwined social and technological innovation, and research and development. Crucial factors may be the transformation of the Internet of Things into the Internet of FAIR data & services. However, Electronic Health Record (EHR) data is in up to 80% unstructured including free text narratives and stored in various inaccessible data warehouses. Enabling the use of data as a driver for learning is challenged by interoperability and reusability.

To address technical needs, key enabling technologies are suitable to convert relevant health data into machine actionable data and to develop algorithms for computerized decision support. To enable data conversions, existing classification and terminology systems serve as definition providers for natural language processing through (un)supervised learning.

To facilitate clinical reasoning and personalized healthcare using LHSs, the development of personomics and functionomics are useful in allied healthcare and nursing. Developing these omics will be determined via text and data mining. This will focus on the relationships between social, psychological, cultural, behavioral and economic determinants, and human functioning.

Furthermore, multiparty collaboration is crucial to develop LHSs, and man-machine interaction studies are required to develop a functional design and prototype. During development, validation and maintenance of the LHS continuous attention for challenges like data-drift, ethical, technical and practical implementation difficulties is required.

**Supplementary Information:**

The online version contains supplementary material available at 10.1186/s12911-023-02372-4.

## Introduction

Transforming health information technologies is critical to safeguard and advance healthcare in a dynamic world. We describe our design for a learning health system (LHS) to aid decision-making in allied health care and nursing. This article is to be viewed as the presentation of a basic theoretical framework that serves as a starting point of a program for the practical design, development and deployment of the LHS for health and healthcare and, in parallel, for the start of a dialogue amongst relevant stakeholders in order to strengthen the framework during this program. We start by drawing attention to the scale of the challenge before discussing the actual development.

With the global challenges and their urgency of the United Nations Sustainable Development Goals [1] in mind, many countries are adopting mission-driven approaches [2, 3]. Missions concerning transformative actions depend on intertwined social and technological innovation and research and development [4]. Transforming from the Internet of Things (IoT) to the Internet of FAIR (Findable, Accessible, Interoperable, Reusable) data & services (IoFAIRaS) is a key factor [5]. This transformation is supported by key enabling technologies [4, 6, 7] such as Life-Science Technologies, Security & Connectivity, Artificial Intelligence, and Foundation Models [8] that were recently put forward. These technologies can be combined in with key enabling methodologies [9] like Critical Design, Fieldlabs and Learning Communities, and Transition Design [4, 9].

The Dutch government introduced a mission-driven approach in 2019 [10]. ‘Health and healthcare’ is one of four nationwide transformative challenges, inspired by five missions of the ministry of health [10–12]. The intended health and healthcare transformation accelerates by the IoFAIRaS-transformation [5] as one of the technological ingredients [4, 7] and Fieldlabs i.e. LHSs [13, 14] as crucial social ingredient to improve personalized health and healthcare [15].

We present a framework that schematically represents the crucial reuse of health and healthcare data to develop a Learning Health System (LHS). A theoretical framework is deemed necessary to be designed, developed and deployed a LHS in a solid and state of the art program [16]. Common components in LHS frameworks are the focus on the LHS, codesign, learning communities, ethics, organization structures, patient outcomes, information technology, security, science, data and performance [16–21]. Here we present a next stage theoretical framework as a mission map and in conjunction with the FAIR principles, key enabling technologies and key enabling methodologies. In our framework we considered each of these components. In general the development of LHS are rapidly evolving though adoption remains difficult [20]. Strong partnership between academic, citizens (patients and relatives), clinical, technical and as well as involving administrative stakeholders in codesign is presented as an important success factor for adoption and implementation of an LHS, whereafter development can start [16, 20–22]. On the other hand organizational culture, adequate data systems and data sharing policies, limited skilled persons, funding and competing priorities remain challenging [18] and, what is more, to be validated in the next steps of our program of design, development and deployment. Our LHS framework focusses on computerized clinical decision support system (cCDSS) for allied healthcare and/or nursing professionals. We explain LHSs in more detail and their importance for the usability of the transformation of health, and healthcare professionals that are embedded in the health system. We also highlight the challenges of using data and data-driven approaches in this context. These challenges might (partly) be overcome by using federated learning data-principles [23–25]. This requires the “FAIRification” of data as this is often inaccessible and unstructured data formats, like in EHR [26, 27].

## Development of learning health system, social, technological and scientific context

**Fig. 1:**
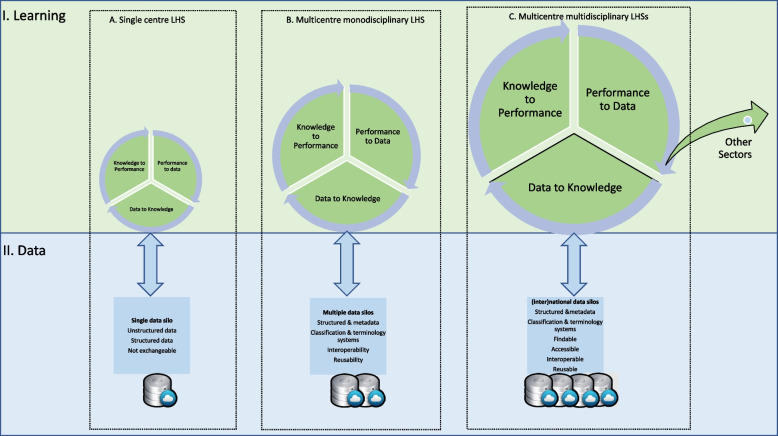
Learning cycle in a Learning Health System

LHSs were introduced as a potential solution to support health and healthcare users and professionals knowledge discovery through learning from clinical data [13, 14, 19]. The learning cycle (Fig. 1, section I) represents an iterative process, that consists of several stages. First; improving users knowledge discovery [28], based on existing data (data to knowledge). For instance, by reflecting on the impact of care delivery or by giving insights in quality of care or cost-effectiveness. Second, learning from data implies the option to utilize the data to improve individuals performance (knowledge to performance) [28], organizations or systems. The third stage is when the improved performance generates new data (performance to data) itself [28]. This accumulation of new data then leads to new knowledge; as a gradual buildup of ‘experience based evidence’ [29–31]. LHS enables users to learn individually and collectively, by reflecting on their own decisions and performances, and on top of this by reflecting on data gathered by others, independent of their location.

To optimize health-related decision making a number of factors are vital, Evidence should be available to the right person, and in the right format, and through the correct channel (e.g. EHR), and at the right time in the workflow [32] using routinely collected and research data [13, 14, 28]. Developing LHSs to optimize health related decision making is made harder due to issues of the accessibility and interoperability of data held in so-called ‘data silos’ (Fig. 1, section II). Data in an EHR is considered as a single data silo, holding both structured and unstructured data formats [26, 27, 33] including free text. This results in locking in the data which restricts the potential learning cycle (Fig. 1, II A). A systematic review of systematic reviews [27] found that EHRs data comprises up to 80% as unstructured including free text narratives. Healthcare registration has become more and more required from clinical and legislation perspectives, and is also paralleled by an exponential increase in digital communication between patients and healthcare providers via online communication portals [27]. EHR contain a variety of nomenclature and languages, abbreviations and definitions and this occurs within and between individuals and within and between health and healthcare disciplines [26, 33, 34]. Using structured (meta)data, standardized terminologies and classifications improves the interoperability and reusability of data. This extends the learning cycle by using multiple data silos (Fig. 1, IIB) and consequently, the global success of LHSs may depend heavily on FAIRification of health and health(care) related data (Fig. 1, IIC). To be able to learn from data collected by others [28], irrespective of location or profession and from multiple decentralized data-silos, data must be FAIR [35–37] preventing numerous amounts of health data exchange between research databases. It should contain not only research and public data, but routinely collected health data as well [13, 14, 28, 38]. The reuse of health data, as the ultimate goal of FAIR, requires a set/system of agreements concerning: standardization of data, metadata, unique identifiers, authentication & authorization, licensing and key infrastructures [35–37].

Developing LHSs for allied health care and nursing demands healthcare insights and innovations that go beyond a disease focused orientation [39]. Clinical reasoning by these professionals, is driven by the appreciation of patient preferences [40, 41] and interrelationship between personal, psychological, social, and environmental determinants [15] and their variability over time, to understand the patients’ functioning and (dis)ability [42, 43]. Besides, these determinants should be the focus in shared decision making, as means to come to personalized healthcare [15, 44].

In the following section we present a framework to construct cCDSS in LHSs taking into account these challenges.

### Framework

#### Constructing computerized clinical decision support in learning health systems

**Fig. 2:**
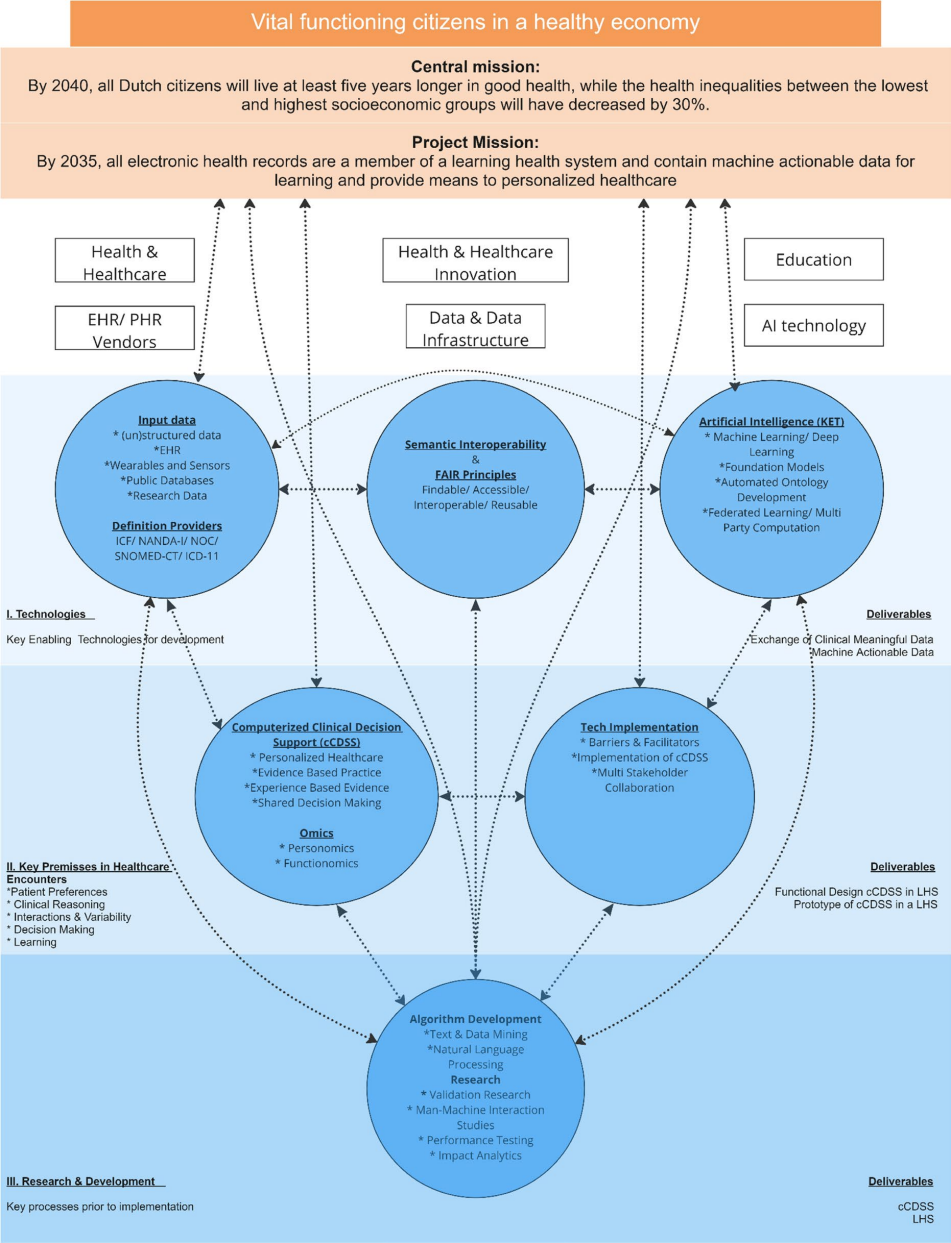
Development of Learning Health system; a mission map

The proposed framework (Fig. 2) uses the Cross Industry Standard Process for Data Mining (CRISP-DM) Extension for Medical Domain [45], in every stage of the development and research. The CRISP-DM is characterized by its iterative nature, where the depth of details of these processes described increases with every cycle [45]. Although multiple data mining models are available, CRISP-DM is feasible and the most commonly used model in the medical domain [46]. Development is not a linear process, but for the sake of clarity in the conceptual description, we present only basic information, divided into technological, healthcare, and research and development aspects.^1^ In addition, we present in supplement 1. in multiple steps (Fig. S1.) the detailed flow for technical development.

### Technologies

Key enabling technologies (KET) [4, 6, 7] to address technical needs are suitable to convert relevant health-related data, from different sources, in machine actionable data [35–37, 47] suitable for clinical meaningful exchange and federated learning [25].

To develop machine actionable data, input data must be transformed into FAIR data [35–37] (Fig. 2-I). Relevant and useful input data is stored in different (in)accessible data silos like EHR systems, public databases, research databases and wearables and sensors. Public databases contain potential useful data for clinical decision making on specific, e.g. environmental, determinants that are not documented in encounters with healthcare professionals. For example, several studies have shown that environmental determinants are potentially relevant determinants of health [39, 48–53]. Automatically linking public data to the EHR is preferable to expecting healthcare professionals to gather this themselves (e.g. In the Netherlands, public data containing clinical useful information on social, environmental and economic determinants, are available in structured format for scientific research by Statistics Netherlands (CBS) [54] and the National Institute for Public Health and the Environment (RIVM)[55]). In addition to linking public and EHR data and transforming routinely collected data into machine actionable data, these procedures should also be performed for empirical research data and wearable sensor data. This IoFAIRaS-transformation, by applying the FAIR principles, maximizes the clinical meaningful reuse of health and healthcare data [35–37] in order to develop multicenter multidisciplinary LHSs as represented in Fig. 1C. Besides the reuse of research data, health data exchange acts [56] demands to put forward health data exchange between health information systems (HIS). The Fast Healthcare Interoperability Resources (FHIR) is the standard to put forward health data exchange between HIS and could speed up the FAIRification of EHR data [38, 57, 58] and data from medical devices as well [59].

With huge amounts of unstructured data collected in EHRs [26], technical and, especially, semantic interoperability remains challenging [60, 61]. Semantic interoperability, defined as the unambiguous representation of clinical concepts [61], is complicated by heterogeneity of data quality and the recognition of concepts of concern in free text narratives suitable for allied healthcare professionals and nurses [60]. To develop technical and semantic interoperable data, all input data, including free text narratives, must be mapped to existing terminology or classification systems using named entity recognition (NER) [62]. Hereto, the International Classification of Function, Disabilities and Health (ICF) [43, 62], NANDA International classification of nursing diagnoses (NANDA-I) [60], Nursing Outcome Classification (NOC), SNOMED-CT [63–65] and International Classification of Diseases (ICD-11) [66] (Fig. 2-I) serve as definition providers as these contain meaningful representations of clinical concepts for allied healthcare professionals and nurses.

When developing and maintaining a LHS with cCDSS, according to data mining models, the data needs to be prepared and modelled [45, 46]. Free text data must be validated, cleaned, repaired and abbreviations must be handled. Subsequently, both structured and unstructured EHR data can be extracted and processed using natural language processing techniques to map them to the classification terminologies [64]. Both unsupervised and supervised learning (i.e. machine learning or deep learning) would be suitable for this (Fig. 2-I). The selection of techniques can be aided by Responsible Technology frameworks like Fundamental Rights and Algorithms Impact Assessment [67].

Respecting the FAIR principles and to prevent transmission of huge amounts of data between silos, the data remains stored in a machine-readable format in its original location [36, 37]. Using Federated learning or Multi Party Computation [23, 24] algorithms are sent to the data without full access to these data (Fig. 2-I). Only the results of processed algorithms are collected preserving the optimum data privacy [23–25].

### Key premises in healthcare encounters

Some systematic reviews [68, 69] have assessed the barriers and factors influencing the implementation of cCDSS. The included studies were limited to technology, organization and healthcare provider perspectives. Using cCDSS affects the primary process of care and, more importantly impacts patients (Fig. 2-II) [70–73]. Recommendations generated by cCDSS aim to improve patient relevant outcomes and therefore facilitate evidence based practice when healthcare professionals discuss these recommendations with their patients [40, 70].

Research has shown that social, functional, environmental and personal determinants for decision making by allied healthcare professionals and nurses [60, 74] are mostly recorded in the unstructured free text areas of EHRs [26]. Within clinical reasoning of allied healthcare professionals and nurses, the ICF [43] and NANDA-I [60] are often used as theoretical knowledge based classifications. These classifications contain social, functional, environmental and personal determinants as elements and can be combined with reasoning frameworks like the hypothesis-oriented algorithm for clinicians II [75, 76], or the nursing process model [77]. While these classifications are useful to describe, clinical concepts are not widely implemented in EHR systems for documentation [62].

For data supported personalized healthcare and precision medicine, development of new, or deployment of existing ontologies are crucial as prerequisite for machine readable data [15, 19, 73, 78, 79]. Personomics [15] and functionomics [42, 80] (Fig. 2-II) in addition to biological omics [81–83] (e.g. genomics, proteomics, metabolomics, etc.) may provide for this [15, 80, 84].

The variety and sequencing of omics is not fully developed and does not cover all domains in health [15]. Interactions between social, psychological, cultural, behavioral and economic factors affecting the patients’ health beliefs and illness approach within the medical system are described as personomics [15]. Studying the complex structure and associations in human functioning has been defined as human functionomics [42, 80]. Personomics and functionomics are suitable for the domain of allied healthcare and nursing, and assisting personalized healthcare provision by these professions [42, 78, 80, 85]. This expands the body of knowledge for decision making, and enables the transformation from a disease focused to a personalized approach.

Transforming the health and healthcare system, in this case, by developing a LHS, requires not only key enabling technologies (KET) but key enabling methodologies (KEM) as well. KET have been proven as international concepts [7, 86], while KEM are limited to national concepts and contain eight methodologies which are currently further developed [4, 9]. A reflection on used KEM will be performed in a later phase of this project. The presence of a LHS with cCDSS, is considered a crucial social ingredient to enable the fulfilment of the missions of the Dutch Ministry of Health to improve health and healthcare quality by learning via clinical data. This evolution affects not only healthcare encounters, but also EHR developers and healthcare organizations [13, 14, 19]. All relevant stakeholders such as; patients, healthcare professionals, data scientists, data engineers, EHR vendors and healthcare organizations must collaborate to identify clinical and technical needs and barriers. Codesign is a crucial element in KEMs [4, 9] and is vital to develop a functional design followed by prototype of a LHS with cCDSS [16, 17, 20–22, 68, 69, 87].

### Future Research & Development

Before deployment in clinical practice, several scientific methods are executed to develop, test and maintain a working LHS with cCDSS (Fig. 2- III). At each stage of development the data is trained and tested on independent datasets until acceptable performance is achieved. Processes are executed with historical data followed by the validation of the results by healthcare professionals and patients before implementation in a real time EHR environment. Research using text and data mining, e,g. natural language processing or deep learning, will be performed to determine the interactions between social, psychological, cultural, behavioral and economic determinants, and human functioning to develop personomics and functionomics.

Man-machine interaction studies are crucial to develop the functional design followed by the prototype of a LHS with cCDSS [88–90]. Supervised learning will be performed for prediction analyses using decision trees, regression analysis and neural networks as analytical tools [83, 91–98]. This lays the framework to develop algorithms suitable for computerized decision support in a LHS. These algorithms, decision rules and the results of the man-machine interaction studies are stepping stones to develop the prototype. It is then essential to assess how well the prototype performs before deployment in clinical research as this saves costs and time [89]. When testing a non-operational system, healthcare professionals enter clinical data into the prototype, test the feasibility, and evaluate whether the cCDSS recommendation is consistent with their clinical expertise and scientific knowledge [99–101]. If the prototype performs acceptably, then an impact analysis of the system precedes implementation in clinical practice. Impact analysis could be done using cluster randomized controlled trials [102–105] or retrospective cohort, pre-post and prospective cohort designs, using a single or multicenter setting [106]. These have been shown to be suitable to evaluate the impact of a cCDSS [102–106]. Multiple baseline studies or interrupted-time-series are also appropriate ways to analyze the impact [107, 108].

### Deliverables

If the processes we have described are followed then EHR providers would be able to convert their data into structured and standardized data. This would make EHR data machine actionable so it can be reused for other purposes. This could be data extraction for quality indicators, or computerized clinical decision support, as described in the literature [34, 109–113].

## General considerations

To achieve the health and healthcare transformation envisaged by the Dutch nationwide transformative challenges we presented a framework to develop a cCDSS as part of a LHS for allied healthcare and nursing. Multiparty collaboration will be crucial to develop, validate and maintain a working LHS [21, 114]. The proposed theoretical framework can also serve as a key enabling methodology [9] to develop and deploy LHSs in other health and healthcare domains and thereafter to be extensively validated and adjusted where necessary. As so, this paper opens up dialogue amongst experts to strengthen our initial thoughts and that of others before and during development of this methodology. Artificial intelligence is a key enabling technology [4, 6, 7] which will be used to develop algorithms for clinical decision support in daily practice. A working LHS with cCDSS could enable personalized healthcare by expanding the learning cycle. The LHS follows the principles of evidence based practice [40] to optimize safe and efficient healthcare provision (knowledge to performance), and enlarge experience based evidence (performance to data) [28–31].

Reusing routinely collected health data could (in accordance with Dutch Electronic Health Data Exchange Act) [56] decrease administrative burden and prevent harmful care [115, 116]. Access to empirical research data or routinely collected health data is impeded by the European General Data Protection Regulation [117, 118]. The development and research of LHSs faces the challenges of data privacy, informed consent and medical ethical approval. Historical or real time data are processed, giving rise to the (im)possibility of informed consent and so approval of medical ethics committees is crucial.

Considering these needs and demands, the FAIRification of health and research data needs to be accelerated. In the era of smart devices and internet of things (IoT) data are a source of information [59] about context, history, physiology, functioning and behavior. Considering the potential to link data from EHRs, empirical research, public data, smart devices and IoT, the internet of FAIR Data & Services facilitates the optimal use of life science technologies and artificial intelligence as key enabling technologies [5–7, 9, 35, 47].

While there are many possible advantages, domain experts, developers and data scientists should be aware of disadvantages. They need to consider aspects like data drift and technical and practical implementation difficulties [119]. First, to overcome these challenges, the data and processed algorithms need to be maintained and tested regularly [120–123]. Second, early multi-stakeholder dialogue and collaboration in a learning community [21] and continuing evaluation of our framework is vital to successfully develop and deploy in clinical care [114, 124, 125]. Third, data sovereignty versus data solidarity [126] will have to be studied. Fourth, beside codesigning via learning communities educational institutes should considerably educate agile health professionals in an agile manner [127].

Patients, nurses and allied healthcare professionals could benefit greatly if we develop and implement learning health systems together. This would improve healthcare and the healthcare system. This roadmap provides guidance on how we could achieve the Dutch and project missions of personalized healthcare via a learning health system.

### Supplementary Information

**Additional file 1. **Development of a Learning Health System; technical flow.

## Data Availability

Not applicable.

## Footnotes

1 Research data, developed ontologies and developed algorithms are considered as open science and therefor will be published in scientific literature, ontology databases like Bioportal, and all algorithms will be made available to EHR providers, other researchers and developers.
