# Supplementary figures and images for: 21st century (clinical) decision support in nursing and allied healthcare. Developing a learning health system: a reasoned design of a theoretical framework
Source: BMC Med Inform Decis Mak. 2023 Dec 5;23:279. doi: 10.1186/s12911-023-02372-4 (PMC10699040; doi:10.1186/s12911-023-02372-4)

Figure S1.


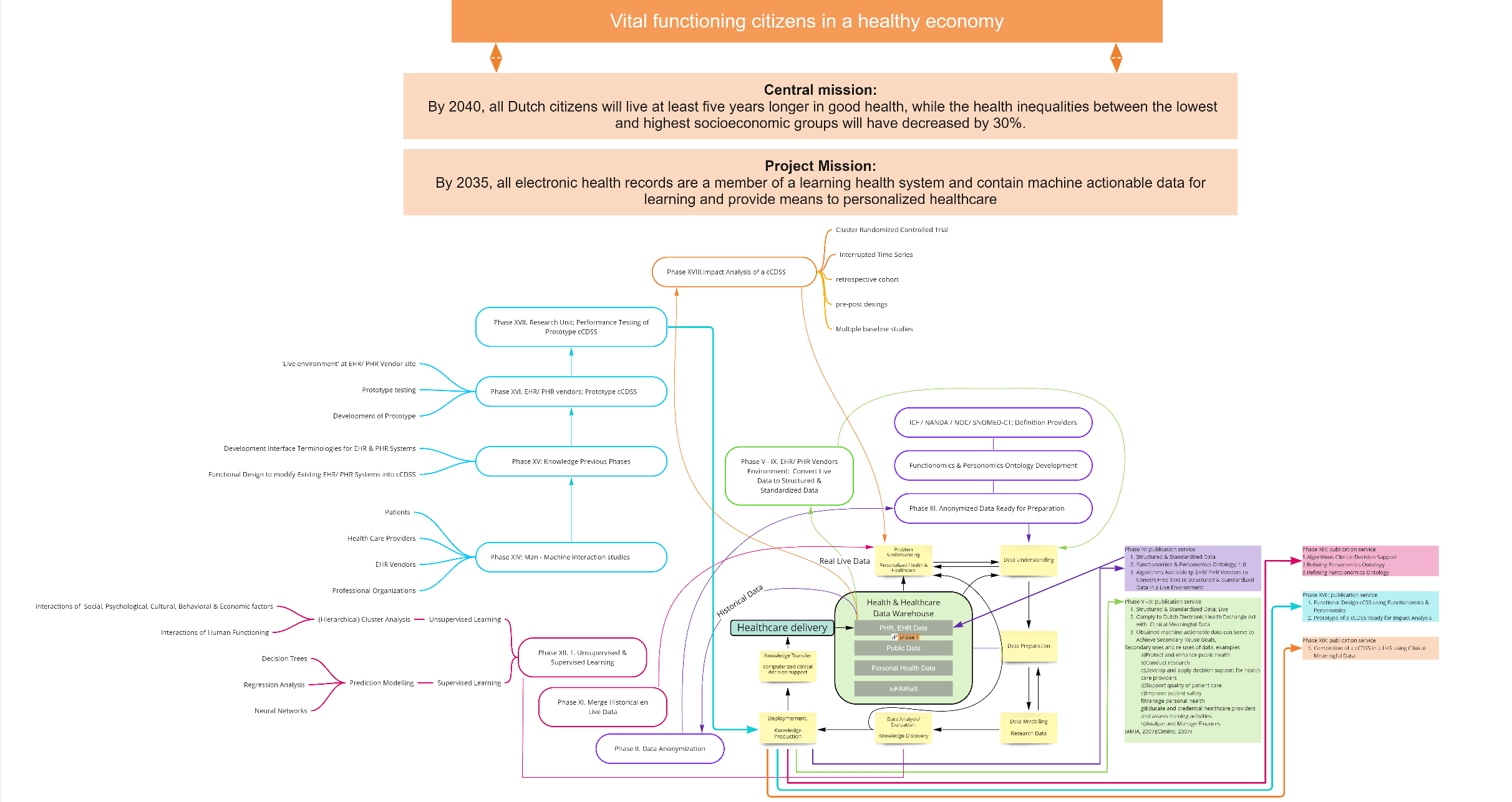


Development of a Learning Health System; technical flow

Supplement: Supplementary file 1 — Additional file 1. Development of a Learning Health System; technical flow. [file 12911_2023_2372_MOESM1_ESM.docx]
